# A Systematic Review of the Placental Translocation of Micro- and Nanoplastics

**DOI:** 10.1007/s40572-023-00391-x

**Published:** 2023-02-27

**Authors:** Eleanor A. Medley, Miranda J. Spratlen, Beizhan Yan, Julie B. Herbstman, Maya A. Deyssenroth

**Affiliations:** 1 Department of Environmental Health Sciences, Mailman School of Public Health, New York, NY 10032 USA; 2 Division of Geochemistry, Lamont-Doherty Earth Observatory of Columbia University, Palisades, NY 10964 USA

**Keywords:** Placenta, Transport, Microplastics, Nanoplastics, Developmental toxicity

## Abstract

**Purpose of Review:**

Despite increasing awareness of the ubiquity of microplastics (MPs) in our environments, little is known about their risk of developmental toxicity. Even less is known about the environmental distribution and associated toxicity of nanoplastics (NPs). Here, we review the current literature on the capacity for MPs and NPs to be transported across the placental barrier and the potential to exert toxicity on the developing fetus.

**Recent Findings:**

This review includes 11 research articles covering in vitro, in vivo, and ex vivo models, and observational studies. The current literature confirms the placental translocation of MPs and NPs, depending on physicochemical properties such as size, charge, and chemical modification as well as protein corona formation. Specific transport mechanisms for translocation remain unclear. There is emerging evidence of placental and fetal toxicity due to plastic particles based on animal and in vitro studies.

**Summary:**

Nine out of eleven studies examined in this review found that plastic particles were capable of placental translocation. In the future, more studies are needed to confirm and quantify the existence of MPs and NPs in human placentas. Additionally, translocation of different plastic particle types and heterogenous mixtures across the placenta, exposure at different periods of gestation, and associations with adverse birth and other developmental outcomes should also be investigated.

**Supplementary Information:**

The online version contains supplementary material available at 10.1007/s40572-023-00391-x.

## Introduction

Since the production of plastics accelerated after the early 1950s, approximately 6300 million metric tons of plastic waste have been generated worldwide, over three-quarters of which have been discarded in various environments [1]. The extensive production and usage of plastic have created an environmental health burden on both macro and micro scales. Large quantities of plastic pollution in natural environments have harmed ecosystems and wildlife, particularly marine life. Increasing amounts of micro- and nanosized plastic particles in water, food, air, and personal care products have put humans at risk of potential adverse health effects as well [2].

### Micro- and Nanoplastic Characteristics

The most commonly used plastic polymers are high- and low-density polyethylene, polyvinyl chloride, polyethylene terephthalate, polypropylene, and polystyrene [3]. Primary plastic particles (or pellets) are produced by industry as the building blocks for nearly every plastic product. They are also created for laboratory purposes, for biomedical applications, and as additives for consumer products such as cosmetics. Secondary plastic particles are formed during weathering processes of larger plastics in natural environments [4]. Historically, there have not been universally agreed upon definitions for the size of microplastics (MPs) and nanoplastics (NPs). MPs are generally classified as < 5 mm in diameter, and NPs are less clearly defined but typically considered to be < 1 µm or < 100 nm in diameter [5, 6, 7]. MPs and NPs exist in a variety of shapes including spherical, angular, irregular, and fibrous forms [8]. These plastic particles can be environmentally transported over long distances and as a result are found in all parts of the world including in Arctic sea ice [9•, 10].

### Sources and Routes of Exposure

Since MPs and NPs are known to be widely present in food, water, and air, humans are most commonly exposed through ingestion and inhalation routes [11]. A common exposure source is bottled water—possibly from both the water itself and its plastic packaging [12]. One evaluation of human consumption estimated that microplastic intake for Americans ranges from 74,000 to 121,000 particles per year [13]. Microplastics were recently detected in human stool, providing evidence of ingestion and gastric exposure [14]. After inhalation or ingestion, MPs and NPs can be taken up in the lungs and gastrointestinal tract by endocytosis and persorption and then enter circulation [11, 15]. Fragments of plastic polymers were recently detected and quantified in human blood for the first time [16•]. More studies are needed to confirm and quantify MPs and NPs in blood and other biological samples.

### Toxicological Implications of Exposure

The toxicity of micro- and nanosized plastic particles likely depends on their physicochemical properties including size, shape, molecular structure, functionalization, surface charge, and material type [8]. There is evidence that exposure to MPs and NPs is associated with inflammation, disruption of immune function, genotoxicity, and oxidative stress in aquatic animals [8, 11, 17]. MPs and NPs can serve as vehicles for other pollutants through sorption processes. For example, endocrine disrupters such as phthalates and bisphenol A, brominated flame retardants, hydrophobic organic contaminants such as polychlorinated biphenyl, and heavy metals can associate with these particles and pose health risks [11, 18, 19, 20]. Furthermore, the surface of MPs and NPs can be rapidly colonized by microbes in the environment, forming biofilms that may impact the microbiome of the host once exposed [11].

### Placental Translocation

**Fig. 1:**
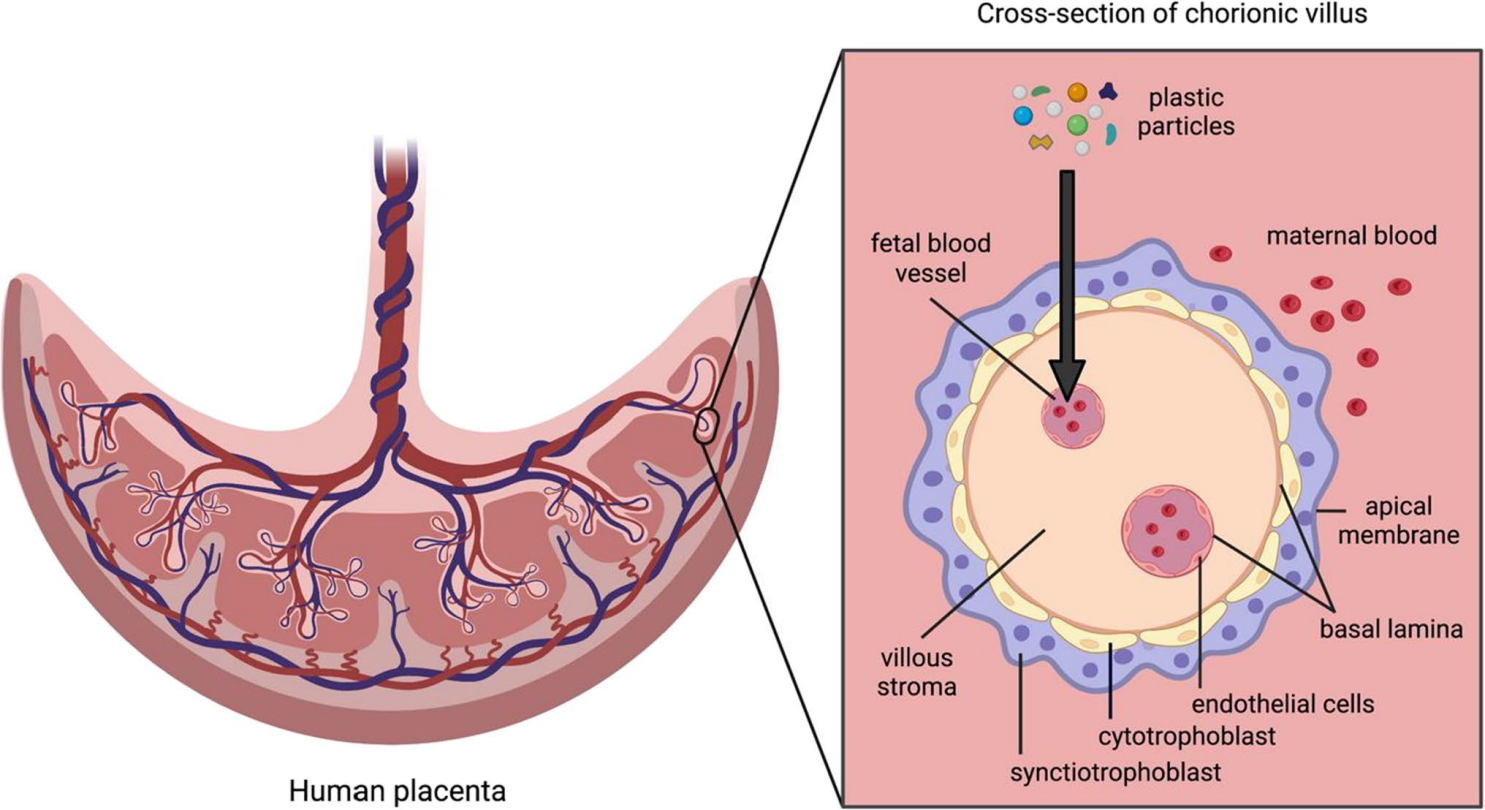
Structure of placental barrier and translocation route for plastic particles. Schematic diagram of human placenta with a labeled cross section of a chorionic villus and arrow showing route for plastic particles to be transported from maternal to fetal circulation. Created with BioRender.com

The potential for wide-ranging health risks associated with MP and NP exposure combined with the heightened susceptibility to environmental agents during fetal development underscores the importance of investigating the maternal-to-fetal transfer of such particles across the placenta. The role of the placenta is to facilitate essential nutrient, gas, and waste exchange for the fetus, but it is not impervious to environmental toxicants. The placental barrier consists of the syncytiotrophoblast layer, cytotrophoblast cells, and the endothelial cell layer of the fetal capillaries (see Fig. 1) [21•]. The syncytiotrophoblast layer is polarized; the basal membrane is in contact with the villous stroma surrounding capillaries on the fetal side and the apical/brush border membrane faces the maternal compartment [21•]. There are four transport mechanisms across the placental barrier: passive diffusion, active transport, phagocytosis/pinocytosis, and biotransformation [22]. The precise mechanism that MPs and NPs might utilize to cross the placental barrier has not yet been elucidated. Various nano-sized materials such as gold, silica, and titanium dioxide have been shown to cross the placental barrier and exert toxicity in animal studies; therefore, placental translocation of at least nanosized plastic particles in humans is of concern [23]. Little is currently known about the existence, concentration, distribution, and types of NPs in the human placenta, largely because existing analytical techniques cannot identify NP particles in complex environmental and biological samples [24].

Existing reviews cover the health risks posed by MPs, NPs, and other nanomaterials including placental transport, but none focus solely on the transport of plastic particles across the placenta [2, 11, 17, 25, 26, 27, 28]. The purpose of this review is to summarize the current literature on the capacity for MPs and NPs to transport across the placental barrier and exert toxicity on the developing fetus in order to identify gaps to be addressed by future research.

## Methods

### Search Strategy

Articles were identified for review through a comprehensive literature search using PubMed and Web of Science databases. The search strategy utilized terms such as “placenta,” “transport,” “particle,” and “plastic” with Boolean operators (see Table S1). The search included study articles published in English with studies conducted in humans and animals. Additionally, references in relevant articles were screened to find eligible publications that were not identified from the initial database searches. The literature search produced articles published between September 1974 and February 2022.

### Selection Criteria

The search targeted human and experimental studies relevant to human health that investigated the translocation of micro- and/or nanosized plastic particles across the placenta. Papers describing findings from in vitro, ex vivo, and in vivo models, as well as observational human studies, were included. Studies investigating developmental toxicity of MPs and NPs in animals that are not placental mammals were excluded. Studies investigating the placental translocation of particles composed of materials other than plastic such as titanium dioxide and silica were excluded.

### Selection of Studies

Reviewers EM and MS independently screened the titles, abstracts, and full text of identified papers to exclude studies that did not fulfill the inclusion criteria. Any discrepancies were discussed until a consensus was reached.

### Synthesis of Results

Selected studies were characterized by particle material type, particle size, dose, exposure duration and route, study model, detection method, and major findings. Studies were then grouped as toxicological or epidemiological evidence, and the former category was evaluated according to reported results for size-dependent transport, charge independent transport, protein corona modulated transport, passive vs. active transport, and distribution and toxicity following transport. We conclude with a qualitative discussion of the study findings regarding the placental translocation of MPs and NPs.

## Results

### Study Selection

**Fig. 2:**
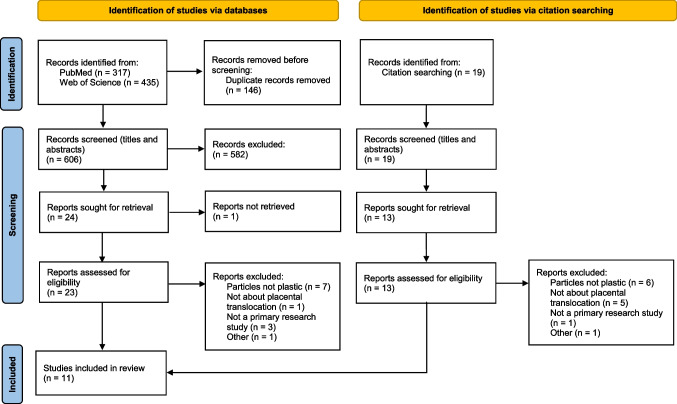
Search process and study selection. From: Page MJ, McKenzie JE, Bossuyt PM, Boutron I, Hoffmann TC, Mulrow CD, et al. The PRISMA 2020 statement: an updated guideline for reporting systematic reviews. BMJ 2021;372:n71. https://doi.org/10.1136/bmj.n71

The literature search was conducted in July 2021 and updated in February 2022. A total of 752 articles were identified using PubMed (*N* = 317) and Web of Science’s Core Collection (*N* = 435). A total of 146 duplicate articles were identified and removed using Endnote. Nineteen additional articles were retrieved from the reference lists of included reviews and other studies. Titles of all papers were screened, abstracts of 51 papers were screened, and 36 full-text papers were assessed for eligibility. Articles were excluded if they did not assess the transport of particles made of plastic across the placental barrier (only investigated the transport of particles made from other materials), if they did not explicitly investigate transport across the placental barrier, or if they were not primary research studies (e.g., review papers or protocols). The final selection of 11 primary research articles included 4 studies using in vitro human cell models, 2 in vivo animal studies, 1 study with both in vitro human cells and an in vivo animal model, 3 studies using ex vivo human placental perfusion models, and 1 observational study in humans. The study selection process is outlined in Fig. 2.

### Study Characteristics

Study characteristics of the existing evidence on the placental translocation of MPs and NPs are summarized in Table 1. The experimental studies reviewed used spherical fluorescent polystyrene NPs ranging from 50 to 500 nm in size with various modifications, charges, and fluorescent labels. The only observational study of human environmental exposure detected MPs in human placenta samples sized 5–10 µm and identified some to be polypropylene. In vitro cell-culture studies used BeWo b30, HPEC-A2, and 3A-Sub-E placental cell lines and primary cell cultures to model the human placental barrier. In vivo and ex vivo studies exposed pregnant mice/rats and human placentas to plastic particles to evaluate translocation. Numerous methods were used to detect the translocation of plastic particles including mass measurements, fluorescence measurements, fluorescence microscopy, confocal microscopy, Raman microspectroscopy, transmission electron microscopy, and asymmetrical flow field-flow fractionation. Some studies also investigated the uptake, distribution, and toxicity of plastic particles in the placenta and fetal tissues and organs.

**Table 1:** Basic characteristics of the studies reviewed

Particle type	Particle diameter	Particle dose	Exposure	Placenta model	Detection method	Major findings	Reference
Fluoresbrite polystyrene latex nanoparticles	50, 100 nm	0.5 mg/mL	24 h time course	In vitro model with BeWo b30 cells	Particle mass measurement, fluorescence measurement, confocal microscopy	- Smaller particles transported to fetal compartment at significantly higher rate - Cellular accumulation of 50-nm particles indicating transcellular transport	Cartwright et al. 2012 [29•]
Positive and negatively charged fluorescent polystyrene nanoparticles, negatively charged fluorescent carboxylated microspheres	50 nm	10 µg/mL	Single dose, 24-h time course	In vitro model with BeWo b30 cells	Fluorescence microplate reader	- Translocation of polystyrene nanoparticles not related to charge and not mediated by known active transporters - Positive NPs induced cytotoxicity of BeWo b30 cells	Kloet et al. 2015 [30•]
Plain fluorescent polystyrene nanoparticles	49, 70 nm	0.5 mg/ml 49 nm NPs, 50 µg/mL 70 nm NPs	Single dose, 24-h time course	In vitro models with BeWo b30 and HPEC-A2 cells	Particle mass measurement, fluorescent microplate reader	Limited translocation of 49-nm polystyrene particles, no translocation of 70-nm polystyrene particles	Aengenheister et al. 2018 [31•]
Rhodamine labeled carboxylated polystyrene particles	50, 500 nm	10, 100 µg/mL	Single dose, 24-h time course	In vitro co-culture model with BeWo b30 and HPEC-A2 cells	Asymmetrical flow field-flow fractionation	- No transport of polystyrene particles across the placental barrier - Polystyrene particles identified as weakly embryotoxic and non-genotoxic	Hesler et al. 2019 [9•]
Fluorescently labeled carboxylate-modified polystyrene beads	20, 40, 100, 200, 500 nm	300 µg	Intravenous injection on embryonic day 17, 4-h time course	In vivo mouse model	Histologic evaluation, HPLC, fluorescence microscopy	- All particle sizes tested observed to cross the placental barrier and distribute in fetal organs in pregnant mice - Uptake of 40 nm particles by 3A-Sub-E trophoblast cells - Cytotoxicity of 20- and 40-nm particles to 3A-Sub-E cells and primary cultures of trophoblasts from term placentas	Huang et al. 2015 [32•]
Carboxylated and PEGlyated fluorochrome labeled polystyrene nanoparticles	50–90 nm	330 µg/mL	Single intravenous injection on 10th and 15th post conception days, 5 min or 4-day time course	In vivo mouse model	Confocal microscopy, spectral imaging fluorescence microscopy	While NPs were detected in the lacunas of the placenta, no particles were found in the embryonic tissues indicating proper barrier function	Kenesei et al. 2016 [33•]
Rhodamine labeled polystyrene beads	20 nm	2.64 × 1014 particles	Intratracheal instillation on gestational day 19, 24-h time course	In vivo rat model	Fluorescent optical imaging, hyperspectral microscopy	Maternal pulmonary exposure to polystyrene nanoparticles results in translocation of nanoplastics to placental and fetal tissues and reduced placental and fetal weight	Fournier et al. 2020 [34••]
Fluorescently labeled polystyrene beads	50, 80, 240, 500 nm	25 µg/mL	180- and 360-min perfusion	Ex vivo human placental perfusion model	Fluorescence microplate reader; transmission electron microscopy	Nanoplastics up to 240 nm in diameter crossed the placental barrier without affecting placenta viability	Wick et al. 2010 [35•]
Fluorescently labeled plain and carboxylated polystyrene beads	50, 240, 300 nm	25 µg/mL	6-h perfusion	Ex vivo human placental perfusion model	Fluorescence microscopy, transmission electron microscopy	- Significant transport of polystyrene particles in the fetal-to-maternal direction with main mechanism likely to be an active, energy-dependent pathway - Accumulation of polystyrene particles in syncytiotrophoblast	Grafmueller et al. 2015 [21]
Fluorescent-labeled plain polystyrene particles	80 nm	40 µg/mL	6-h perfusion	Ex vivo human placental perfusion model	Fluorescence microplate reader, transmission electron microscopy	Dynamically forming protein corona significantly influenced transfer of particles across placenta	Gruber et al. 2020 [36•]
Pigmented microplastic fragments, some identified as polypropylene	5–10 µm			Observational study of human placentas	Raman microspectroscopy	Microplastics found in fetal side, maternal side, and chorioamniotic membranes in 4 out of 6 human placentas tested	Ragusa et al. 2021 [37••]

While some studies used multiple experimental conditions to investigate both placental translocation and toxicology of plastic particles, study characteristics (e.g., particle dose, exposure, placenta model, and detection method) are provided for the translocation studies specifically. The reported “major findings” include results from toxicological investigations as well (Table 1).

### Size-Dependent Transport

Using an ex vivo human placental perfusion model, Wick et al. (2010) observed size-dependent maternal-to-fetal placental translocation of fluorescent polystyrene particles. After 180 min of perfusion, fluorescence measurements and transmission electron microscopy micrographs showed that beads sized 50, 80, and 240 nm were able to cross the placenta to the fetal compartment, while 500-nm beads did not [35•]. Leveraging an in vitro placental cell culture model, Cartwright et al. (2012) seeded BeWo b30 cells on polycarbonate membrane Transwells and observed that 50-nm fluoresbrite polystyrene particles were transported to the fetal compartment at a sixfold higher rate than 100-nm particles, also indicating size-dependent transport of NPs. Confocal microscopy showed cellular accumulation of 50-nm particles, indicating transcellular transport [29•]. Further supporting size-dependent transport, Aengenheister et al. (2018) found limited placental translocation of 49-nm polystyrene particles and no translocation of 70-nm polystyrene particles in BeWo b30 cells as well as the microvascular human placental venous endothelial cell line HPEC-A2. In an in vivo mouse model, Huang et al. (2015) found polystyrene particles ranging 20–500 nm could cross the placental barrier. Placental uptake and trophoblast internalization was greatest for 40-nm particles, and there was no clear linear association between particle size and uptake [32•].

### Charge Independent Transport

Using BeWo b30 cells, Kloet et al. (2015) observed that translocation of 50-nm polystyrene nanoparticles was not dependent on the surface charge of the particles. Translocation of three types of 50-nm polystyrene NPs, one positively charged and two negatively charged, was assessed [30•]. Particles gained a similar negative charge in medium with 10% fetal calf serum. After 24 h of incubation, the positively charged NP and one type of negatively charged NP showed a time-dependent increase in the basolateral compartment [30•]. Though the two types of negatively charged NPs were approximately the same size and charge, they showed significantly different translocation across the BeWo b30 model [30•]. Because the two NPs were produced by different manufacturers, the authors hypothesized the difference in translocation observed could be attributed to unknown differences in the chemical groups on the surface of the particles [30•]. Potentially consistent with this theory, Grafmueller et al. (2015) found increased placental translocation of plain polystyrene beads compared with carboxylate-modified beads of the same size. Overall, these findings suggest that the translocation of plastic particles across the placenta is determined by a combination of factors, including particle size and the presence of chemical modifications.

### Protein Corona Modulated Transport

It is plausible that the interaction of plastic particle surfaces with various endogenous biomolecules affects particle transport. In biological fluids, proteins can rapidly cover the surface of nanomaterials forming a *protein corona.* Using the ex vivo human placental perfusion model, Gruber et al. (2020) found that dynamic protein coronas influenced the translocation of plain 80-nm polystyrene nanoparticles across the placental barrier. First, the group showed the addition of plasma to the perfusion medium increased the maternal-to-fetal transfer of NPs [36•]. The composition of the protein coronas also changed substantially in both relative abundance and quantity [36•]. For example, they detected a significant increase in the number of proteins on NPs that were in contact with the placenta versus in plain medium and a significant decrease in the number of proteins on NPs in fetal circulation versus maternal circulation [36•]. Using shotgun proteomics, they identified albumin (HSA) and immunoglobulin G (IgG) as major proteins of the NP coronas [36•]. Media supplemented with HSA showed a higher maternal-to-fetal transfer rate than media supplemented with IgG; however, the final NP concentrations in maternal circulation were similar for both media [36•]. In all media tested including the control, Gruber et al. (2020) observed placental translocation of NPs which suggests any protein corona–facilitated transport is also accompanied by another baseline mechanism.

### Passive vs. Active Transport

Kloet et al. (2015) also investigated the role of specific transport mechanisms by assessing whether the distribution of NPs in the compartments changed in the presence of inhibitors of endocytosis and specific ATP-binding cassette transporters. They found that inhibitors of P-glycoprotein and breast cancer resistance protein had no effect on translocation of either NP and that an inhibitor of multidrug resistance protein 1 increased basolateral translocation of the positively charged NP and had no effect on the translocation of the negatively charged NP [30•]. Overall, they concluded that inhibition of these specific active transport mechanisms did not significantly affect NP translocation and so translocation occurs preferentially by passive diffusion [30•].

In contrast, Grafmueller et al. (2015) posit that the translocation of polystyrene particles across the placenta mainly involves an active, energy-dependent transport pathway. The investigators used the ex vivo human placental perfusion model to assess the bidirectional transfer of both plain and carboxylate modified polystyrene particles with fluorescent labels 50–300 nm in diameter [21•]. After 6 h of perfusion, bidirectional translocation of NPs up to 300 nm was observed and was higher in the reverse (fetal-to-maternal) direction. This finding suggests that there are different transport mechanisms on the fetal and maternal sides of the placenta [21]. Antipyrine, a small molecule used as a marker of passive transport, reached equilibrium after 4–6 h of perfusion but concentration equilibrium of the NPs was not reached [21]. Furthermore, while antipyrine translocation in the fetal-to-maternal direction was delayed due to lower exchange surface on the fetal side, the translocation of NPs in this direction was enhanced [21]. Taken together, these observations suggest that translocation of NPs occurs by an energy-dependent mechanism. The authors proposed phagocytosis or endocytosis as possible translocation mechanisms rather than specific transporters; primary human macrophages have been reported to phagocytose 100-nm polystyrene nanoparticles [21, 38]. Additionally, both Grafmueller et al. (2015) and Gruber et al. (2020) observed the accumulation of polystyrene NPs in the syncytiotrophoblast layer.

### Toxicity and Distribution

Since there is accumulating evidence that plastic particles can cross the placental barrier, it is important to investigate the toxic potential of these particles. In the studies using ex vivo placental models by Grafmueller et al. (2015) and Wick et al. (2010), general measures of placental viability, functionality, and transfer were observed not to be affected by perfusion of plastic particles [21•, 35•]. Hesler et al. (2019) used BeWo b30 cells to conduct a multi-endpoint toxicological assessment of 50- and 500-nm carboxy-modified polystyrene particles in vitro. Consistent with Wick et al. (2010) and Grafmueller et al. (2015), this study did not observe any adverse effects of NP exposure on placental barrier integrity as measured by transepithelial electrical resistance [9•]. Exposure to 50-nm and 500-nm particles was associated with a slight increase in viability [9•]. As the carboxyl groups conferred a negative charge to the polystyrene particles, this finding aligns with the previously discussed study by Kloet et al. (2015) in which they found only positively charged NPs induced cytotoxicity of BeWo b30 cells.

In different in vitro models, the human 3A-Sub-E trophoblast cell line and villous cytotrophoblasts derived from term placentas of normal pregnancies, Huang et al. (2015) did observe cytotoxicity of carboxylate-modified NPs. Following exposure to 20- and 40-nm particles in both trophoblast models, they observed elevated cleaved caspase 3, a marker of apoptosis induction, based on Western blot analysis [32•]. They also observed reduced cell proliferation after exposure to 20-nm nanoparticles in both types of trophoblast models through analysis of BrdU incorporation [32•].

Interestingly, Hesler et al. did not observe transport across a placental BeWo b30/HPEC-A2 co-culture model at either concentration tested (10 and 100 µg/mL) after 24 h of exposure. The researchers used a different detection system than previous studies, asymmetrical flow field-flow fractionation (AF4), and noted the possibility of translocated particles being present below the limit of detection [9•]. Using confocal microscopy, however, the researchers observed internalization of the polystyrene particles by placental cells so they pursued studies of embryotoxicity [9•]. An embryonic stem cell test in a mouse cell line, mES-D3, showed weak embryotoxicity of the polystyrene particles [9•]. Through assessment of micronuclei formation and p53 expression after exposure to polystyrene particles, the study concluded the particles were non-genotoxic at the concentrations tested (0.1–10 µg/mL) [9•].

Given some evidence of the toxicity of plastic particles on trophoblast and embryonic cell lines, the distribution of plastic particles in fetal organs after transport across the placenta warrants consideration. Kenesei et al. (2016) used an in vivo model to investigate the distribution of surface-modified fluorescent polystyrene nanoparticles after intravenous injection of pregnant mice. Using spectral imaging fluorescence microscopy, they found carboxylated NPs in the lacunas of the placenta 5 min after particle administration [33•]. Particles were completely cleared from the placenta after 4 days, and NPs were not observed in embryonic tissues, indicating proper barrier function [33•]. However, Fournier et al. (2020) detected the distribution of 20-nm rhodamine-labeled polystyrene beads in fetal tissues after intratracheal instillation of pregnant rats in late gestation. Elevated fluorescence and NPs were observed in the fetal placenta, liver, lung, kidney, heart, and brain using fluorescent optical imaging and hyperspectral darkfield microscopy, suggesting that NPs can translocate from the maternal lungs to a variety of fetal tissues through the placenta [34••]. Similarly, Huang et al. (2015) found that carboxylate-modified polystyrene particles 20–500 nm crossed the mouse placenta and distributed in the fetal brain, lungs, and liver. Huang et al. (2015) found no evidence of tissue architecture alteration, degeneration, necrosis, or inflammatory cell infiltration in these organs via histopathological evaluation [32•]. However, Fournier et al. (2020) observed reduced placental weight and fetal weight 24 h after exposure, indicating plastic particle exposure during pregnancy could affect birth outcomes.

### Environmental Exposure in Humans

In a pilot observational study, Ragusa et al. (2021) were the first to report MPs found in human placenta samples, confirming the placental translocation of plastic particles observed in ex vivo and in vitro models. The researchers obtained placentas from 6 patients in Italy with uneventful pregnancies [37••]. Samples taken from the maternal side, fetal side, and chorioamniotic membranes were analyzed for the presence of MPs by Raman microspectroscopy [37••]. To avoid contamination, plastic-free delivery and sample preparation protocols were used [37••]. Twelve pigmented MPs ~ 5 and 10 µM in size were detected in the placentas of 4 women and found in all 3 types of tissues tested [37••]. While a much larger sample size would be needed to make conclusions about the distribution of MPs in placentas at the population level, the discovery of MPs in the fetal side of human placental samples warrants further investigation. This study detected significantly larger particles (MPs) than investigated in the previously discussed studies (NPs). Given the evidence of size-dependent transport of plastic particles across the placental barrier in various models, evidence of environmental MPs crossing the placental barrier during pregnancy in humans suggests that smaller-sized NPs are also likely to do so. However, Ragusa et al. (2021) were not able to detect NPs due to the challenges of detecting small plastic particles that are not fluorescently labeled and resolution limitations of traditional Raman microscopy.

## Discussion

Eleven studies investigating the capacity for MPs and NPs to cross the placental barrier were reviewed. Placental translocation of plastic particles was observed in multiple experimental models including in vitro cell cultures, in vivo animal models, and ex vivo human placental perfusion systems as well as in an observational study of MPs in human placentas after delivery. One study conducted in an in vitro co-culture context and one study conducted in mice did not observe placental translocation of the plastic particles tested. Given the wide variety of systems used, it is important to weigh the strengths and limitations of the different models when comparing study results.

While in vitro models are easier to obtain and utilize for high-throughput studies, they can be over-simplified compared to an in vivo environment. Promisingly, translocation of compounds across in vitro models has been shown to correlate well with the ex vivo human placental perfusion model [29•, 39, 40]. Cartwright et al. (2012) and Kloet et al. (2015) both used BeWo b30 trophoblast cells, an in vitro model derived from gestational choriocarcinoma of the fetal placenta grown on transwell membranes separating apical and basolateral compartments. Though the model does not represent the complete placental environment, trophoblast cells serve as the rate-limiting barrier [29•]. In their optimization of the in vitro Transwell trophoblast model, Cartwright et al. (2012) highlight the importance of avoiding adherence of the NPs to the system’s Transwell matrix and ensuring monolayer formation. Citing the absence of the barrier function of placental endothelial cells in such models, Aengenheister et al. (2018) characterized an in vitro co-culture model with BeWo b30 cells and HPEC-A2 placental microvascular endothelial cells. This co-culture model was utilized in the study by Hesler et al. (2019) in which no placental transport of 50- and 500-nm carboxy-modified polystyrene particles was observed. Transformed cells may not accurately represent normal placental physiology. Though BeWo b30 cells are most commonly used in placental translocation studies, it has been suggested that the first-trimester human trophoblast cell line ACH-3P may actually mimic transport results from ex vivo perfusion models better and more closely resemble primary cells [41]. Huang et al. (2015) utilized primary villous cytotrophoblasts isolated from placentas after delivery [32•].

In vivo studies of placental translocation of plastic particles in a whole-body system are valuable for understanding more realistic and comprehensive exposure conditions. As such, the study Fournier et al. (2020) conducted in rats provides insight into fetal deposition of NPs after maternal inhalation. Additionally, Kenesei et al. (2016) and Huang et al. (2015) investigated placental translocation of NPs in mice. However, extrapolation of findings from animal studies to humans is difficult due to significant interspecies differences in the placenta; rats show differences in placental morphology, yolk sac function, and steroid hormone biosynthesis, for example [42, 43].

The ex vivo human placenta model used by Wick et al. (2010), Grafmeuller et al. (2015), and Gruber et al. (2020) provides advantages over animal models by more accurately representing the structure of the human placental barrier and over cell culture models by allowing for dynamic flow during exposure. The process of tissue degradation, however, limits experiments with these ex vivo models to a few hours [35•]. Because placentas must be obtained from patients after delivery, the model can be difficult to access and exclusively represents late gestation when the thickness of the placental barrier is reduced to allow increased maternal–fetal exchange [35•]. Findings by Gruber et al. (2020) using the ex vivo placental perfusion model highlight the importance of the biological fluid environment in affecting protein corona formation on particles and subsequent placental transfer. Experimental studies of the placental transport of plastic particles use commercially purchased plastic beads, which can have a wider size distribution than reported by the manufacturer. In their article about artifacts in transfer studies of NPs using the ex vivo human placenta perfusion model, Grafmueller et al. (2015) discuss the challenges of obtaining monodisperse suspensions of polystyrene nanoparticles and minimizing loss of fluorescent dye intensity from the particles, though these issues could affect translocation studies conducted in other models as well [44]. It may be important to consider how accurately monodisperse suspensions of plastic particles represent real-world exposures in light of evidence of biogenic aggregation of MPs in marine environments [45, 46]. Overall, it is noteworthy that, despite the aforementioned limitations, all three studies conducted with the ex vivo human placenta perfusion model and the observational study of human placentas after delivery showed evidence of placental translocation of plastic particles. Ragusa et al. (2021) reported using a plastic-free protocol, but potential contamination from plastic equipment still poses a significant challenge for studying environmental exposures to MPs and NPs.

## Conclusions and Future Directions

The current literature suggests that the placental translocation and toxicity of MPs and NPs depend on physicochemical properties such as size, charge, and chemical modification. Wick et al. (2010), Cartwright et al. (2012), and Aengenheister et al. (2018) showed size-dependent transport of polystyrene particles across the placenta, while Kloet et al. (2015) found that polystyrene particles of the same size had different translocation properties likely dependent on functional groups. Kloet et al. (2015) also showed that functional groups and charge affect the cytotoxicity of polystyrene particles on placental trophoblast cells. This has implications for the risk assessment of the developmental toxicity of MPs and NPs—more characteristics modulating the translocation and toxicity of plastic particles justify the assessment of more variations and types. Furthermore, Gruber et al. (2020) showed dynamic protein coronas affect placental translocation of NPs, posing another variable in experimental risk assessment.

Nine out of eleven studies examined in this review found that plastic particles were capable of placental translocation; observations of MPs in human placentas by Ragusa et al. (2021) make this consensus even more convincing. The mechanism that plastic particles use to cross the placenta remains unclear: Kloet et al. (2015) suggested it was passive diffusion and Grafmueller et al. (2015) suggested it was active transport. It is possible that these mechanisms are not mutually exclusive and the process depends on some combination of both.

All of the experimental studies utilized mostly uniform, spherical, polystyrene particles. Polystyrene is widely used to package foods and has been found to be one of the most frequently detected plastic polymers in human blood [16•]. MPs and NPs can be composed of a variety of polymers, though they exist in a variety of shapes, and contain numerous additives. Given the diversity of findings using highly controlled particles, it will be necessary to investigate other types and heterogenous mixtures more representative of the totality of environmental exposures. Only one study reviewed (Ragusa et al., 2021) evaluated placental transport of plastic particles from the environment, highlighting the need to further investigate more realistic human exposures. Furthermore, most of the studies reflected exposure during late gestation, emphasizing the need for more future studies to investigate whether placental translocation and developmental toxicity of MPs and NPs change when exposure occurs earlier in pregnancy. One study found that injection of nano-sized amine- and carboxyl-modified polystyrene particles into the extraembryonic tissue of mouse embryos on day 7.5 of gestation inhibits growth, supporting the need for more investigation into developmental toxicity throughout gestation [47•].

Given the evidence that MPs and NPs can not only translocate across the placenta but also subsequently deposit in fetal tissues and exert toxicity [9•, 32•, 34••], it will be important to investigate whether maternal exposure to plastic particles is associated with adverse birth and other longer-term developmental outcomes in humans. It has been reported that maternal exposure to 5-µm polystyrene MPs during gestation and lactation in mice altered metabolism in F1 and even F2 generations [48•]. Additional studies are warranted to investigate whether this developmental toxicity occurs in humans and elucidate whether it is a direct effect from transport across the placenta or indirectly from maternal effects [49]. Finally, the role of maternal susceptibility factors affecting exposure and response to MPs and NPs, including diet and genetics, should also be investigated. Exposure to environmental agents during fetal development can have critical developmental consequences that affect health outcomes throughout the life course. Given the persistence and ubiquity of plastic in the environment, further research is essential to better understand how this omnipresent material is affecting human health from the very start of development.

## Supplementary Information

Below is the link to the electronic supplementary material.

Supplementary file1 (DOCX 17 KB)

## References

[1] Geyer R, Jambeck JR, Law KL (2017). Production, use, and fate of all plastics ever made. Sci Adv.

[2] Kannan K, Vimalkumar K (2021). A review of human exposure to microplastics and insights into microplastics as obesogens. Front Endocrinol (Lausanne).

[3] Li WC, Tse HF, Fok L (2016). Plastic waste in the marine environment: a review of sources, occurrence and effects. Sci Total Environ.

[4] Duis K, Coors A (2016). Microplastics in the aquatic and terrestrial environment: sources (with a specific focus on personal care products), fate and effects. Environ Sci Eur.

[5] Gigault J (2018). Current opinion: what is a nanoplastic?. Environ Pollut.

[6] ter Halle A, Ghiglione JF (2021). Nanoplastics: a complex, polluting Terra Incognita. Environ Sci Technol.

[7] Hartmann NB (2019). Are we speaking the same language? Recommendations for a definition and categorization framework for plastic debris. Environ Sci Technol.

[8] Stapleton PA (2019). Toxicological considerations of nano-sized plastics. AIMS Environ Sci.

[9] • Hesler M, et al. “Multi-endpoint toxicological assessment of polystyrene nano- and microparticles in different biological models in vitro,” ToxicolVitr. 2019;61:104610. **Found weak embryotoxicity of polystyrene particles in mouse cell line.** 10.1016/j.tiv.2019.104610 31362040

[10] Lusher AL, Tirelli V, O’Connor I, Officer R (2015). Microplastics in Arctic polar waters: the first reported values of particles in surface and sub-surface samples. Sci Rep.

[11] Wright SL, Kelly FJ (2017). Plastic and human health: a micro issue?. Environ Sci Technol.

[12] Mason SA, Welch VG, and Neratko J. “Synthetic polymer contamination in bottled water,” Front Chem. 2018;6. 10.3389/fchem.2018.00407. 10.3389/fchem.2018.00407 PMC6141690 30255015

[13] Cox KD, Covernton GA, Davies HL, Dower JF, Juanes F, Dudas SE (2019). Human consumption of microplastics. Environ Sci Technol.

[14] Schwabl P (2019). Detection of various microplastics in human stool: a prospective case series. Ann Intern Med.

[15] Rothen-Rutishauser B, Mühlfeld C, Blank F, Musso C, Gehr P (2007). Translocation of particles and inflammatory responses after exposure to fine particles and nanoparticles in an epithelial airway model. Part Fibre Toxicol.

[16] • Leslie HA, van Velzen MJM, Brandsma SH, Vethaak D, Garcia-Vallejo JJ, Lamoree MH. Discovery and quantification of plastic particle pollution in human blood. Environ Int. 2022;2021:107199. **First quantification of plastic particles in human blood.** 10.1016/j.envint.2022.107199 35367073

[17] Prata JC, da Costa JP, Lopes I, Duarte AC, Rocha-Santos T (2020). Environmental exposure to microplastics: an overview on possible human health effects. Sci Total Environ.

[18] Amato-Lourenço LF, dos Santos Galvão L, de Weger LA, Hiemstra PS, Vijver MG, Mauad T (2020). An emerging class of air pollutants: potential effects of microplastics to respiratory human health?. Sci Total Environ.

[19] Wardrop P, et al. “Chemical pollutants sorbed to ingested microbeads from personal care products accumulate in fish,” Environ Sci Technol. 2016;50(7):4037–4044. 10.1021/acs.est.5b06280 26963589

[20] Magadini DL, Goes JI, Ortiz S, Lipscomb J, Pitiranggon M, Yan B (2020). Assessing the sorption of pharmaceuticals to microplastics through in-situ experiments in New York City waterways. Sci Total Environ.

[21] • Grafmueller S, et al. Bidirectional transfer study of polystyrene nanoparticles across the placental barrier in an ex vivo human placental perfusion model. Environ Health Perspect. 2015;123(12):1280–6. **Observed significant transport of polystyrene particles in the fetal-to-maternal direction.** 10.1289/ehp.1409271 PMC4671239 25956008

[22] Syme MR, Paxton JW, Keelan JA (2004). Drug transfer and metabolism by the human placenta. Clin Pharmacokinet.

[23] Yamashita K (2011). Silica and titanium dioxide nanoparticles cause pregnancy complications in mice. Nat Nanotechnol.

[24] Brachner A (2020). Assessment of human health risks posed by nano-and microplastics is currently not feasible. Int J Environ Res Public Health.

[25] Sripada K, Wierzbicka A, Abass K, Grimalt JO, Erbe A, Röllin HB (2022). A children’s health perspective on nano- and microplastics. Environ Health Perspect.

[26] Bongaerts E, Nawrot TS, Van Pee T, Ameloot M, Bové H (2020). Translocation of (ultra)fine particles and nanoparticles across the placenta; a systematic review on the evidence of in vitro, ex vivo, and in vivo studies. Part Fibre Toxicol.

[27] Ferrante MC, Monnolo A, Del Piano F, Raso GM, Meli R (2022). The pressing issue of micro- and nanoplastic contamination: profiling the reproductive alterations mediated by oxidative stress. Antioxidants.

[28] Mortensen NP, Johnson LM, Grieger KD, Ambroso JL, Fennell TR (2019). Biological interactions between nanomaterials and placental development and function following oral exposure. Reprod Toxicol.

[29] • Cartwright L, et al. “In vitro placental model optimization for nanoparticle transport studies,” Int J Nanomedicine. 2012;7:497–510. **Observed size-dependent transport and cellular accumulation.** 10.2147/IJN.S26601 PMC3273982 22334780

[30] • Kloet SK, et al. “Translocation of positively and negatively charged polystyrene nanoparticles in an in vitro placental model,” Toxicol Vitr. 2015;29(7):1701–1710. **Observed different transport properties of negatively charged particles, cytotoxicity of positively charged particles**. 10.1016/j.tiv.2015.07.003 26145586

[31] • Aengenheister L, et al. “An advanced human in vitro co-culture model for translocation studies across the placental barrier,” Sci Rep. 2018;8(1):1–12. **Observed size-dependent transport.** 10.1038/s41598-018-23410-6 PMC5876397 29599470

[32] • Huang JP, et al. “Nanoparticles can cross mouse placenta and induce trophoblast apoptosis,” Placenta. 2015;36(12):1433–1441. **Observed distribution of particles in fetal organs of mice, cytotoxicity of particles.** 10.1016/j.placenta.2015.10.007 26526105

[33] • Kenesei K, Murali K, Czéh Á, Piella J, Puntes V, and Madarász E. “Enhanced detection with spectral imaging fluorescence microscopy reveals tissue- and cell-type-specific compartmentalization of surface-modified polystyrene nanoparticles,” J Nanobiotechnol. 2016;14(1):1–14. **Particles not observed in embryonic tissues of mice.** 10.1186/s12951-016-0210-0 PMC4936314 27388915

[34] Fournier SB (2020). Nanopolystyrene translocation and fetal deposition after acute lung exposure during late-stage pregnancy. Part Fibre Toxicol.

[35] • Wick P, et al. “Barrier capacity of human placenta for nanosized materials,” Environ Health Perspect. 2010;118(3):432–436. **Observed size-dependent transport.** 10.1289/ehp.0901200 PMC2854775 20064770

[36] • Gruber MM, et al. “Plasma proteins facilitates placental transfer of polystyrene particles,” J Nanobiotechnol. 2020;18(1):1–14. **Observed dynamic formation of protein coronas affecting particle transport.** 10.1186/s12951-020-00676-5 PMC7487953 32907583

[37] Ragusa A (2021). Plasticenta: first evidence of microplastics in human placenta. Environ Int.

[38] Lunov O (2011). Differential uptake of functionalized polystyrene nanoparticles by human macrophages and a monocytic cell line. ACS Nano.

[39] Li H, Van Ravenzwaay B, Rietjens IMCM, Louisse J (2013). Assessment of an in vitro transport model using BeWo b30 cells to predict placental transfer of compounds. Arch Toxicol.

[40] Poulsen MS, Rytting E, Mose T, Knudsen LE (2009). Modeling placental transport: correlation of in vitro BeWo cell permeability and ex vivo human placental perfusion. Toxicol Vitr.

[41] Rothbauer M, Patel N, Gondola H, Siwetz M, Huppertz B, Ertl P (2017). A comparative study of five physiological key parameters between four different human trophoblast-derived cell lines. Sci Rep.

[42] Furukawa S, Tsuji N, Sugiyama A (2019). Morphology and physiology of rat placenta for toxicological evaluation. J Toxicol Pathol.

[43] Schmidt A, Morales-Prieto DM, Pastuschek J, Fröhlich K, Markert UR (2015). Only humans have human placentas: molecular differences between mice and humans. J Reprod Immunol.

[44] Grafmueller S (2015). Transfer studies of polystyrene nanoparticles in the ex vivo human placenta perfusion model: key sources of artifacts. Sci Technol Adv Mater.

[45] Drago C, Pawlak J, Weithoff G (2020). Biogenic aggregation of small microplastics alters their ingestion by a common freshwater micro-invertebrate. Front Environ Sci.

[46] Michels J, Stippkugel A, Lenz M, Wirtz K, and Engel A. “Rapid aggregation of biofilm-covered microplastics with marine biogenic particles,” Proc R Soc B Biol. Sci. 2018;285:1885. 10.1098/rspb.2018.1203 PMC6125906 30158309

[47] Tian F (2009). Surface modification and size dependence in particle translocation during early embryonic development. Inhal Toxicol.

[48] Luo T, Wang C, Pan Z, Jin C, Fu Z, Jin Y (2019). Maternal polystyrene microplastic exposure during gestation and lactation altered metabolic homeostasis in the dams and their F1 and F2 offspring. Environ Sci Technol.

[49] Dugershaw BB, Aengenheister L, Hansen SSK, Hougaard KS, Buerki-Thurnherr T (2020). Recent insights on indirect mechanisms in developmental toxicity of nanomaterials. Part Fibre Toxicol.

