# Supplementary material for: A Systematic Review of the Placental Translocation of Micro- and Nanoplastics
Source: Curr Environ Health Rep. 2023 Feb 27;10(2):99–111. doi: 10.1007/s40572-023-00391-x (PMC10300151; doi:10.1007/s40572-023-00391-x)
Supplement: Supplementary file 1 — Supplementary file1 (DOCX 17 KB) [file 40572_2023_391_MOESM1_ESM.docx]

**Supplementary Materials**

**Table S1.** Search Strategy

| **Database** | **Search** | **Additional Settings** | **No. of Results** | **Date Range** |
| --- | --- | --- | --- | --- |
| Pubmed | ((“placenta”[MeSH Terms] OR “placent*”[Title/Abstract] OR “fetus”[MeSH Terms] OR “fetal”[Title/Abstract]) AND (“translocat*”[Title/Abstract] OR “transfer”[Title/Abstract] OR “transport”[Title/Abstract] OR “pass*”[Title/Abstract] OR “distribut*”[Title/Abstract] OR “detect*”[Title/Abstract] OR “presen*”[Title/Abstract] OR “cross”[Title/Abstract]) AND (“particl*”[Title/Abstract] OR “nanoparticl*”[Title/Abstract] OR “microparticl*”[Title/Abstract]) AND (“poly*”[Title/Abstract] OR “plastic*”[Title/Abstract] OR “nanoplastic*”[Title/Abstract] OR “microplastic*”[Title/Abstract])) | English language  Abstract, full text availability  Studies in humans, other animals | 317 | Sept 1974 - Jan 2022 |
| Web of Science Core Collection [v.5.35] | (TS = (“placent*”) OR TS = (“fetal”) OR TS = (“fetus”)) AND (TS = (“translocat*”) OR TS = (“transfer”) OR TS = (“transport”) OR TS = (“pass*”) OR TS = (“distribut*”) OR TS = (“detect*”) OR TS = (“found”)) AND (TS = (“particl*”) OR TS = (“nanoparticl*”) OR TS = (“microparticl*”)) AND (TS = (“poly*”) OR TS = (“plastic*”) OR TS = (“nanoplastic*”) OR TS = (“microplastic*”)) | English language  Document type: article | 435 | Jan 1991 - Feb 2022 |
